# Long-term dynamics of hematological data and spleen volume in cirrhotic patients after liver transplantation-various dynamics depending on etiology

**DOI:** 10.1186/2193-1801-2-374

**Published:** 2013-08-07

**Authors:** Masatoshi Ishigami, Yoji Ishizu, Yasuharu Onishi, Hideya Kamei, Tetsuya Kiuchi, Akihiro Itoh, Yoshiki Hirooka, Yoshiaki Katano, Hidemi Goto

**Affiliations:** Department of Gastroenterology and Hepatology, Nagoya University School of Medicine, 65 Tsuruma-cho, Showa-ku, 466-8550 Nagoya, Japan; Department of Transplant Surgery, Nagoya University School of Medicine, Nagoya, Japan

**Keywords:** Hematological parameters, Spleen volumes, Liver transplantation, Long-term dynamics

## Abstract

**Background:**

Hypersplenism is a common complication in cirrhotic patients, and liver transplantation would be one of the effective treatments. However, detailed dynamics, especially over a long term, are not fully understood. We investigated the long-term dynamics of hematological data and spleen volumes, as well as their correlation in cirrhotic patients who underwent liver transplantation.

**Patients and methods:**

We studied 53 cirrhotic patients who underwent liver transplantation at our institute and followed for more than 1 year. Hematological data were collected from medical records, while spleen volumes were determined by CT volumetry at 0, 1, 3, 6, 12, 24, 36, 48, 60 postoperative months (POM).

**Results:**

(1) Platelet (Plt) and hemoglobin (Hb) levels were gradually increased up to 18 and 10 POM, respectively, in contrast with white blood cells (WBC), which remained mostly unchanged from pretransplantation levels. (2) Spleen volume was sharply decreased in the first POM, then showed a slower but steady decline up to 48 POM. (3) Spleen volume was significantly correlated with hematological data, though the levels were generally weak (Plt: r = 0.433, p < 0.001; Hb: r = 0.233, p < 0.001; WBC: r = 0.217, p = 0.001). (4) Spleen volume was strongly correlated with all hematological parameters in HBV patients (Plt: r = 0.617, p < 0.0001; Hb: r = 0.401, p < 0.001; WBC: r = 0.387, p < 0.001), in contrast with that in other etiologies, which had generally weak correlations though some were statistically significant.

**Conclusions:**

We investigated the long-term dynamics of hematological data and spleen volume in cirrhotic patients after liver transplantation. Unique dynamics and correlations between them were found among the different etiologies investigated.

## Introduction

Hypersplenism and pancytopenia are common complications in patients with liver cirrhosis, which occasionally induce life-threatening complications or loss of quality of life (QOL) from bleeding, infection, or anemic symptoms. The incidence of hypersplenism among cirrhotic patients varies from 15–70% (Yanaga et al. 1989; Mutchnik et al. 1980).

Among the various hematological conditions in these patients, thrombocytopenia is well-studied, though its mechanism remains a matter of debate even in the recent days. Traditionally, sequestration of platelets by the spleen has been proposed as the main mechanism, other mechanisms such as reduced thrombopoietin or decreased survival time of platelet have been recently considered to contribute to these mechanisms (Pradella et al. 2011; Witters et al. 2008). Nevertheless, it has not been well elucidated to what extent hypersplenism and splenic sequestration contribute to the mechanism of thrombocytopenia in cirrhotic patients.

For rescuing patients from these disorders, direct removal of spleen by splenectomy (Puttini et al. 1979; Soper & Rikkers 1982), volume reduction of spleen by partial splenic embolization (Stiegler et al. 1998; Sangro et al. 1993; Herrero et al. 1997), or portal decompression by transjuglar intrahepatic portosystemic shunt (Alvarez et al. 1996; Pursnani et al. 1997) are performed as the effective methods in these days. However, some controversial reports have noted that thrombocytopenia may persist even after splenectomy or partial decompression (Pradella et al. 2011; Sanyal et al. 1996; Gschwantler et al. 1999).

Liver transplantation can also be an effective treatment by replacing cirrhotic liver with normal one and thereby reducing portal hypertension and consequently diminishing splenomegaly. In fact, some reports have noted that thrombopoiesis occurred soon after liver transplantation (Yanaga et al. 1989; Stiegler et al. 1998; Borel Rinkes et al. 1991; Peck-Radosavljevic et al. 2000) and spleen volumes were significantly lowered at 1-2 postoperative months as compared with the preoperative levels (Yanaga et al. 1989).

In those studies, platelet dynamics were observed for only very short periods (14 days to 2 months), and data for long-term dynamics are scarce. Moreover, finding regarding white blood cells (WBC) and red blood cells (RBC) in cirrhotic patients are particularly lacking and found only in older studies (Holzbach et al. 1964).

In the present study, we investigated the long-term dynamics of hematological data and spleen volume after liver transplantation that is not known well in cirrhotic patients. In addition, we also analyzed the differences in dynamics among different etiologies.

## Patients and methods

### Patients

We performed liver transplantation for 84 cirrhotic patients between 2003 and 2011 at our institution. Among them, following patients were excluded; 10 who died within one year, 3 who were followed for less than 1 year after transplantation, 7 who were treated with IFN which affects hematological data, 5 who underwent splenectomy, 6 for whom we were unable to measure precise spleen volume because artifacts caused by splenic arterial coiling. Finally, 53 patients were included in this study and their background data are shown in Table 1. Among these, patients were divided into 4 groups based on etiologies; Hepatitis B virus (HBV), Hepatitis C virus (HCV), Primary Biliary Cirrhosis (PBC), and others (5 Primary Screlosing Cholangitis (PSC), 5 cholestatic except PBC or PSC, 3 Autoimmune Hepatitis (AIH), 1 Wilson’s disease, and 1 undetermined etiology) classified as nonBnonC (NBNC) because of the small numbers of each etiology. And among them, 27 patients reached 5 years for follow-up time, so we also did the long-term analysis in these patients. Median follow up times were 76 months in 27 patients in long-term analysis and 60 months in all 53 patients in this study, and follow up period in each subgroup was included in Table 1.

**Table 1:** **Patients’ backgrounds**

	All	HBV	HCV	PBC	NBNC
n	53	19	6	13	15
Recipient Age (years old)	19-61(52)	40-61(53)	55-61(57)	27-58(52)	19-55(32)
Recipient Sex (M/F)	30/23	16/3	5/1	0/13	9/6
Donor Age (years old)	20-60(33)	20-60(34)	24-54(26.5)	21-60(33)	25-54(32)
Donor Sex (M/F)	30/23	8/11	5/1	7/6	10/5
Creatinine (mg/dl)	0.25-13.90(0.70)	0.50-13.90(0.80)	0.60-1.20(0.80)	0.40-1.00(0.63)	0.25-1.48(0.64)
Total Bilirubin (mg/dl)	0.3-41.0(4.4)	0.5-8.4(2.3)	1.0-18.5(1.9)	2.1-41.0(13.2)	0.3-37.3(10.2)
PT-INR	1.01-3.34(1.49)	1.15-3.04(1.63)	1.29-2.63(1.47)	1.24-2.18(1.54)	1.01-3.34(1.39)
MELD score	8.4-29.2(17.7)	9.0-27.4(16.6)	10.7-12.7(12.9)	11.5-29.2(19.6)	8.4-26.7(18.2)
Follow up period	13-104(60)	15-104(47)	45-78(71.5)	13-98(51)	16-97(60)

*HBV* Hepatitis B virus, *HCV* Hepatitis C virus, *PBC* Primary Biliary Cirrhosis, *NBNC* nonB, nonC, *M* Male, *F* Female, *PT*-*INR* Prothrombin Time-International Normalized Ratio.

Data are shown as minimum-maximum range, with median in parenthesis.

### Hematological data and spleen volumes

White blood cell (WBC) counts, hemoglobin (Hb) levels, and platelet (Plt) counts were picked up from medical records in preoperatively, every postoperative month until 12 postoperative months (POM), and then every 3 months until 60 POM. Finally, data at 1253 time points were collected from 53 patients.

Spleen volume was determined based on digital data of follow-up computed tomography (CT) in preoperatively, 1, 3, 6, 12 POM and then annually until 60 POM and collected retrospectively. They were approximated as the sum of truncated polygonal pyramids from 5-mm thin slices using Aquilion multislice CT system (Toshiba Medical Systems, Tokyo, Japan). Thus, CT data at 278 time points were collected from 40 patients.

### Statistical analysis

For comparisons of data that consisted of continuous numbers among the groups, Student’s t-test was applied. To analyze correlations between spleen volume and hematological data, linear regression analysis was applied. p < 0.05 in each analysis was considered as statistically significance. For linear regression analysis, correlation coefficients were used to evaluate the impact of the correlations. All analyses were done with SPSS 16.0 J software (SPSS Japan, Tokyo, Japan).

## Results

### Short-term (1 year) and long-term (5 years) dynamics of hematological data for each hematological subset

**Figure 1:**
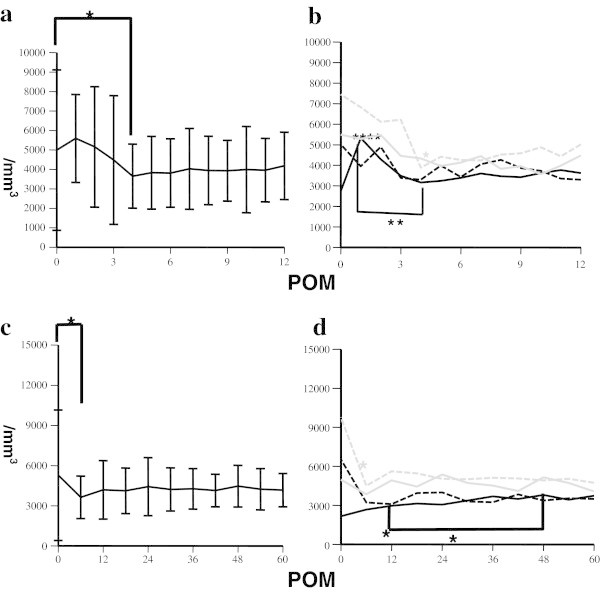
**Short term and long term dynamics of WBC after liver transplantation. a**. Short-term (1 year) dynamics of WBC after liver transplantation. *p < 0.05 for comparison between baseline level and at 4 POM. **b**. Short-term (1 year) dynamics of WBC in different etiologies after liver transplantation. Black closed line: HBV, black broken line: HCV, gray closed line: PBC, gray broken line: NBNC, ***(black asterisk) p < 0.001 in comparison between baseline WBC levels and those at 1 POM in HBV, **(black asterisk) p < 0.01 for comparison between WBC levels at 1 POM and those at 4 POM in HBV, *(gray asterisk) p < 0.05 in comparison between WBC levels at baseline and at 5 POM in NBNC. **c**. Long-term (5 years) dynamics of WBC after liver transplantation. *p < 0.05 for comparison between WBC levels at baseline and at 6 POM. **d**. Long-term (5 years) dynamics of WBC among different etiologies after liver transplantation. Black closed line: HBV, black broken line: HCV, gray closed line: PBC, gray broken line: NBNC *(black asterisk) p < 0.05 for comparison between WBC levels at baseline and at 12 POM, and between those at 12 POM and 48 POM in HBV, *(gray asterisk) p < 0.05 for comparison between WBC levels at baseline and at 6 POM in NBNC.

**Figure 2:**
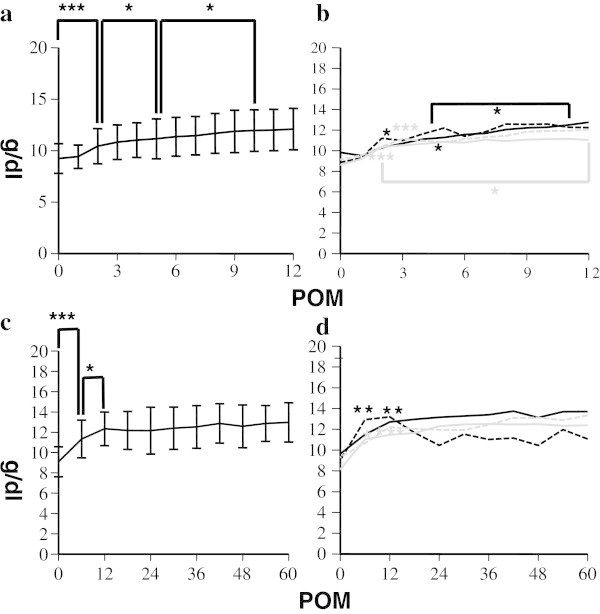
**Short term and long term dynamics of Hb after liver transplantation. a**. Short-term (1 year) dynamics of Hb after liver transplantation. *p < 0.05 in comparison between Hb levels at 2 POM and 5 POM, and 5 POM and 10 POM, ***p < 0.001 for comparison between Hb levels at baseline and at 2 POM. **b**. Short-term (1 year) dynamics of Hb among different etiologies after liver transplantation. Black closed line: HBV, black broken line: HCV, gray closed line: PBC, gray broken line: NBNC, *(black asterisk) p < 0.05 in comparison between Hb levels at baseline and at 5 POM and 5 POM and 11 POM in HBV, and Hb levels at baseline and 2 POM in HCV, ***(gray asterisk) p < 0.001 for comparison between Hb levels at baseline and 2 POM in PBC, and 3 POM in NBNC, *(gray asterisk) p < 0.05 for comparison between Hb levels at 2 POM and 12 POM in PBC. **c**. Long-term (5 years) dynamics of Hb after liver transplantation. Black closed line: HBV, black broken line: HCV, gray closed line: PBC, gray broken line: NBNC, *p < 0.05 for comparison between Hb levels at 6 POM and 12 POM, ***p < 0.001 for comparison between Hb levels at baseline and 6 POM. **d**. Long-term (5 years) dynamics of Hb among different etiologies after liver transplantation. **(black asterisk) p < 0.01 for comparison between Hb levels at baseline and 6 POM in HCV, and 12 POM in HBV, *(gray asterisk) p < 0.05 for comparison between Hb levels at baseline and 6 POM in PBC, ***(gray asterisk) p < 0.001 for comparison between Hb levels at baseline and 12 POM in NBNC.

**Figure 3:**
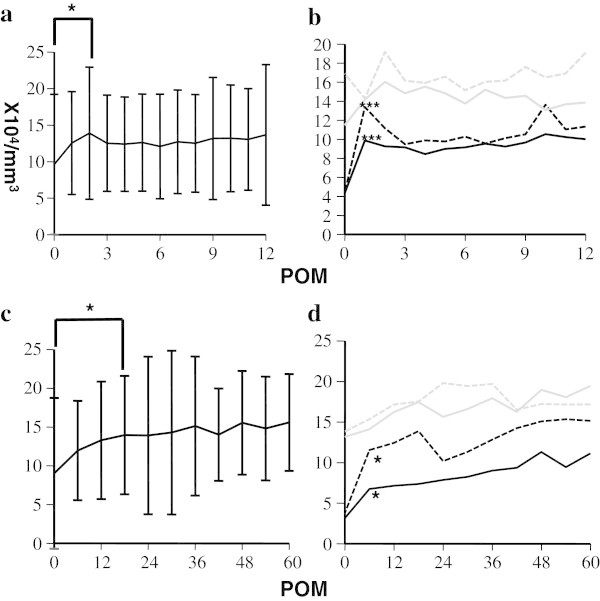
**Short term and long term dynamics of Plt after liver transplantation. a**. Short-term (1 year) dynamics of Plt after liver transplantation. *p < 0.05 in comparison between Plt levels at baseline and 2 POM. **b**. Short-term (1 year) dynamics of Plt among different etiologies after liver transplantation. Black closed line: HBV, black broken line: HCV, gray closed line: PBC, gray broken line: NBNC, ***(black asterisk) p < 0.001 in comparison between Plt levels at baseline and 1 POM in HBV and HCV. **c**. Long-term (5 years) dynamics of Plt after liver transplantation. *p < 0.05 in comparison between WBC levels at baseline and 18 POM. **d**. Long-term (5 years) dynamics of Plt among different etiology after liver transplantation. Black closed line: HBV, black broken line: HCV, gray closed line: PBC, gray broken line: NBNC, *(black asterisk) p < 0.05 in comparison between Plt levels at baseline and 6 POM in HBV and HCV.

The short-term dynamics of WBC were shown in Figure 1a. Those counts increased to peak at 1 POM, though the changes were not statistically significant as compared with baseline levels. Thereafter, they significantly decreased to 4 POM and reached a plateau, and this trend remained unchanged in long-term analysis (Figure 1b). In contrast, Hb levels increased more slowly up to 10 POM, then reached a plateau and remained there for the long terms (Figure 2a, 2c). Platelet counts were significantly increased at 2 POM, and then slightly decreased (Figure 3a), after which they seemed to increase gradually, though the statistical impact was relatively small. Platelet levels at 18 POM and later were significantly higher only when compared to the baselines (Figure 3c). These data showed that hematological parameters reached a plateau faster than expected, though some differences were seen among the various etiologies.

### Short-term (1 year) and long-term (5 years) dynamics of hematological data among different etiologies

Figure 1b shows the short-term dynamics of WBC counts among different etiologies. Those in HBV patients sharply increased at 1 POM as compared with baseline, then significantly decreased at 4 POM and reached a plateau. In contrast, those for NBNC patients significantly decreased at 4 POM as compared with baseline and reached a plateau. As for PBC and HCV, there were no significant changes in the short-term period. Over the long-term, those trends were unchanged except in HBV patients, in whose WBC levels at 48 POM were higher as compared to 12 POM (Figure 1d). Hb levels were higher at 2 POM in HCV patients, and 3 POM in NBNC patients as compared with baselines then both reached a plateau thereafter (Figure 2b, 2d). In PBC patients, that was higher at 2 POM as compared with baseline, and continued to gradually rise with the level at 12 POM, which was significantly higher as compared with that at 2 POM, and after which they reached a plateau. In HBV patients, Hb level increased more slowly, with that at 5 POM higher than that of the baseline, and gradually rising until level at 11 POM which was significantly higher than that at 5 POM after which they reached a plateau (Figure 2b, 2d). As for platelet counts, patients with viral hepatitis (HBV and HCV) showed sharply increased levels up to 3 POM and until reaching a plateau, which was in contrast to non-viral patients (PBC and NBNC) whose levels were generally unchanged (Figure 3b and 3d).

These data showed that dynamics of each hematological parameter varied among the different etiologies of liver cirrhosis after liver transplantation.

### Dynamics of spleen volume after liver transplantation in cirrhotic patients

After liver transplantation, theoretically hypersplenism is expected to be cured because of resigning from liver fibrosis. We next analyzed the dynamics of spleen volume after liver transplantation in all patients and among the different etiologies.

**Figure 4:**
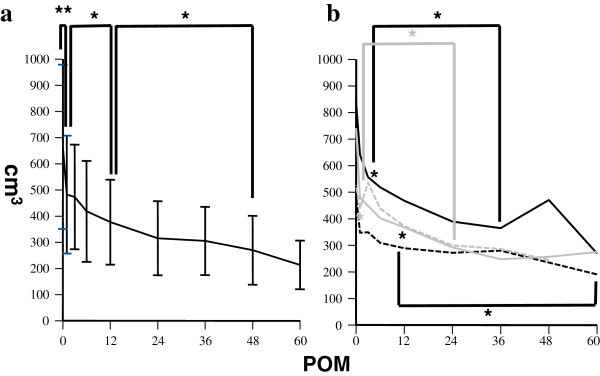
**a. Dynamics of spleen volumes after liver transplantation.** **p < 0.01 in comparison between spleen volumes at baseline and 1 POM. *p < 0.05 in comparison between spleen volumes at 1 POM and 12 POM, and 12 POM and 48 POM. **b**. Dynamics of spleen volumes among different etiologies after liver transplantation. Black closed line: HBV, black broken line: HCV, gray closed line: PBC, gray broken line: NBNC, *(black asterisk) p < 0.05 in comparison between spleen volumes at baseline and 3 POM, and 3 POM and 36 POM in HBV, and those at baseline and 12 POM, and 12 POM and 48 POM in HCV. *(gray asterisk) p < 0.05 in comparison between spleen volumes at baseline and 1 POM, and 1 POM and 24 POM in PBC.

Spleen volumes showed a slow, but steady and significant decrease at least until 48 POM (Figure 4a). The differences regarding dynamics among the different etiologies are shown in Figure 4b. In viral patients, spleen volumes showed a steady decrease over the long term, while in patients with PBC, a sharp decline was seen at 1 POM then a slower decrease occurred up to 24 POM. In contrast, there were no significant changes within 60 months after liver transplantation in the NBNC patients.

These findings show that spleen volumes generally had a steady and significant decrease in our cirrhotic patients after liver transplantation except in those with NBNC.

### Correlations between spleen volume and hematological data in all patients and among different etiologies

**Figure 5:**
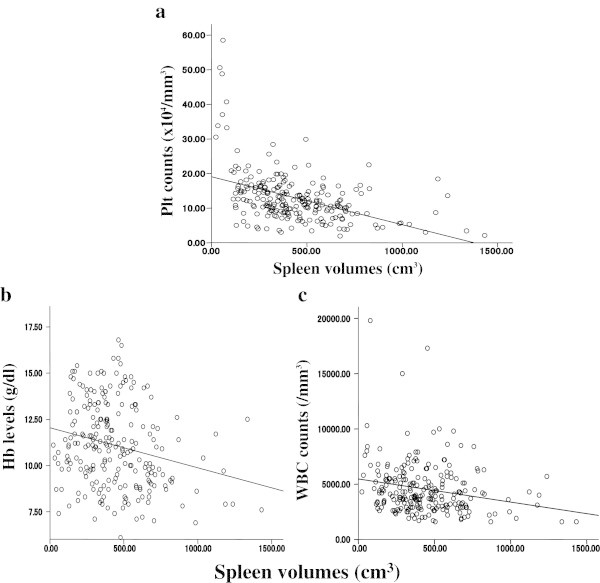
**Correlations between spleen volumes and each hematological parameter after liver transplantation. a**. Correlation between Plt count and spleen volume, r = 0.433, p < 0.001, **b**. Correlation between Hb level and spleen volume, r = 0.233, p < 0.001, **c**. Correlation between WBC count and spleen volume, r = 0.217, p = 0.001.

Figure 5 shows correlations among hematological data and spleen volume. Significant correlations were seen between spleen volume and each hematological data point, though the correlations were generally weak (Plt: r = 0.433, p < 0.001; Figure 5a, Hb: r = 0.233, p < 0.001; Figure 5b, WBC: r = 0.217, p = 0.001; Figure 5c).

**Figure 6:**
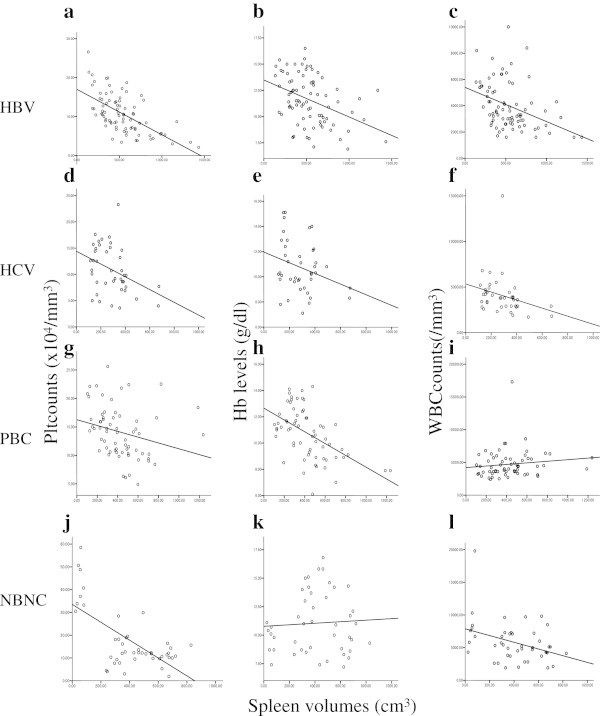
**Correlations between spleen volumes and each hematological parameter among different etiologies after liver transplantation. a**. Correlation between Plt count and spleen volume in HBV, r = 0.617, p < 0.001, **b**. Correlation between Hb level and spleen volume in HBV, r = 0.401, p < 0.001, **c**. correlation between WBC count and spleen volume, r = 0.387, p < 0.001, **d**. Correlation between Plt count and spleen volume in HCV, r = 0.364, p < 0.001, **e**. Correlation between Hb level and spleen volume in HCV, r = 0.278, p = 0.079, **f**. Correlation between WBC count and spleen volume in HCV, r = 0.279, p = 0.078, **g**. Correlation between Plt count and spleen volume in PBC, r = 0.256, p = 0.09, **h**. Correlation between Hb level and spleen volume in PBC, r = 0.279, p < 0.001, **i**. Correlation between WBC count and spleen volume in PBC, r = 0.128, p = 0.314, **j**. Correlation between Plt count and spleen volume in NBNC, r = 0.660, p < 0.001, **k**. Correlation between Hb level and spleen volume in NBNC, r = 0.056, p = 0.715, **l**. Correlation between WBC count and spleen volume in NBNC, r = 0.316, p = 0.013.

Analysis in each etiology, relatively strong correlations were seen in HBV patients, with the correlations between platelets and spleen volumes the strongest (Plt: r = 0.617, p < 0.001; Figure 6a, Hb: r = 0.401, p < 0.001; Figure 6b, WBC: r = 0.387, p < 0.001; Figure 6c, respectively). In other etiologies, the correlations were generally weak or not significant (Figure 6d-6i, 6k, 6l) except that between Plt level and spleen volume in NBNC (r = 0.660, p < 0.001, Figure 6j).

These results suggest that changes in spleen volume were significantly correlated with those of hematological data, though the correlations were generally weak. Therefore, there may be some independent and undefined mechanisms other than hypersplenism related to the changes in hematological data during the progression of cirrhosis and recovery after liver transplantation (Peck-Radosavljevic et al. 2000).

## Discussion

The reduction in number of hematological cells in cirrhotic patients has already been shown to recover after liver transplantation in the previous studies by others (Yanaga et al. 1989; Stiegler et al. 1998; Borel Rinkes et al. 1991; Peck-Radosavljevic et al. 2000). However, those studies focused on the relatively short-term dynamics of platelets, while those over a long term and for red and white blood cells have not been well understood. Recently, Ohira, et.al. reported fine and detailed investigation of the correlations between spleen volume and all three hematological parameters, though even this report investigated up to 6 months after liver transplantation (Ohira et al. 2009).

In the present study, we studied the dynamics of hematological data and spleen volume in 53 patients with cirrhosis who underwent liver transplantation without a splenectomy. Each hematological parameter showed different dynamics with platelet and WBC showing faster increases with peak levels reached at 1, and 2 POM, respectively (Figures 1a, 1c and 3a, 3c). Thereafter, WBC level soon returned to the baseline (Figure 1a and 1c), whereas platelets only slightly decreased and then rose again slowly until 18 POM (Figure 3a, 3c). The dynamics of Hb were slower as recovery continued for about 1 year and then reached a plateau (Figure 2a, 2c).

Over the long term dynamics, spleen volume showed a slow, but steady and statistically significant decrease until at least 48 POM (Figure 4a) as compared with the long-term dynamics of hematological data which showed nearly no changes in the later phase (Figures 1c, 2c and 3c). Spleen volume and each hematological parameter were significantly correlated with each other, though the correlations were generally low (Figure 5a, 5b and 5c). These findings suggest hypersplenism to be one of the main causes of pancytopenia in cirrhotic patients, though its impact is smaller than expected. Thus, other independent mechanisms might also contribute for this disease formation.

Regarding platelets, relatively large numbers of candidates have been investigated in the previous studies. The most studied factor is thrombopoietin, a cytokine produced by the liver that regulates megakaryocyte maturation and platelet production (Pradella et al. 2011; Witters et al. 2008). Adinolfi, et al showed that hepatic fibrosis and altered production of thrombopoietin are central players in the pathogenesis of thrombocytopenia in patients with chronic viral hepatitis without splenomegaly (Adinolfi et al. 2001). Moreover, thrombopoietin has been shown to rapidly increase after liver transplantation (Peck-Radosavljevic et al. 2000). However, those studies presented relatively short-term data, and the long-term effects of this cytokine remain largely unknown.

In addition to the platelet production, decreased platelet survival time may be a mechanism related to thrombocytopenia in cirrhotic patients (Pradella et al. 2011; Witters et al. 2008). Thrombocytopenia in cirrhotic patients has been reported to have a remarkable homology with idiopathic thrombocytopenic purpura as the reticulated platelet proportion (proportion of young platelets) and glycocalicin index (marker of platelet production) were significantly higher in both diseases as compared with healthy controls (Witters et al. 2008; Kajihara et al. 2003; Kajihara et al. 2007). Pradella et al. showed platelet-associated antibodies and anti-platelet antibodies were significantly higher in HCV-positive patients as compared to other etiologies of cirrhosis (Pradella et al. 2011). In this study, the correlation between platelet level and spleen volume was relatively weak (p = 0.364, Figure 6f) in HCV. Thus, in addition to spleen sequestration, decreased survival may be one of the likely mechanisms for thrombocytopenia in HCV-positive cirrhotic patients, though direct evidence for platelet related antibodies was not investigated in this study.

In contrast to the other etiologies investigated, the correlation between spleen volume and platelet count in HBV patients was strong (p = 0.617, Figure 6c). Thus, splenic sequestration was considered to be the main mechanism, in contrast with PBC patients, in whom spleen volume was relatively large though the correlation between spleen volume and platelet count were weak, and NBNC patients, in whom the correlation was high, while spleen volumes was generally unchanged. Our study revealed these varing dynamics among the different etiologies even when only the correlation between platelet count and spleen volume were investigated.

As for WBC and Hb levels in cirrhotic patients, information was much more scant. Sequestration of RBC in the spleen was shown to occur according to portal pressure and spleen size in an old study. Jiao et al. reported that erythrocyte creatine, which is considered to be a marker of destruction of erythrocytes, showed good correlations with spleen size and reticulocytes (Jiao et al. 2001). Therefore, erythrocytes may be supposed to be correlated with spleen volume better than other hematological parameters.

In our study, Hb was shown to increase slower than the other hematological parameters, thus the impact of spleen volume, which showed a steady decrease, might be supposed to affect the erythrocyte count to a larger degree than other parameters. On the other hand, an unknown mechanism might also exist because Hb level reached a plateau at 10 POM (Figure 2a and 2c), and the correlation between Hb and spleen volume was relatively weak (Figure 5b). As for WBC, data are quite rare. In the present study, WBC were increased at 1 POM and then decreased to the baseline or lower, while correlation between spleen volume and WBC was much weaker as compared to other parameters (Figure 5c).

## Conclusions

In summary, we investigated the long-term dynamics of hematological parameters and spleen volume in cirrhotic patients after liver transplantation. Each hematological parameter increased in the early postoperative period, and thereafter, though each generally reached a plateau in the later phase. In contrast in spleen volume, a steady reduction was found up to 48 POM. Hematological data and spleen volume were significantly correlated with each other, though their correlations were generally weak. Furthermore, these dynamics were very different among the etiologies for cirrhosis investigated. To the best of our knowledge, this is the first report to investigate long-term dynamics after liver transplantation. From these findings, we intend to further investigate the detailed mechanisms of these differences among etiologies, as well as discrepancies between hematological data and spleen volume.

### Consent and ethics of this study

Written informed consent was obtained from the patient for the publication of this report and any accompanying images and this study has done according to the guidance of Helsinki Declaration.
